# Prevalence of common chronic disease and multimorbidity patterns in Guangdong province with three typical cultures: analysis of data from the Diverse Life-Course Cohort study

**DOI:** 10.3389/fpubh.2023.1163791

**Published:** 2023-05-04

**Authors:** Yaoda Hu, Huijing He, Qiong Ou, Jing Nai, Li Pan, Xingming Chen, Ji Tu, Xuejun Zeng, Guo Pei, Longlong Wang, Binbin Lin, Qihang Liu, Guangliang Shan

**Affiliations:** ^1^Department of Epidemiology and Biostatistics, Institute of Basic Medical Sciences, Chinese Academy of Medical Sciences/School of Basic Medicine, Peking Union Medical College, Beijing, China; ^2^Department of Sleep Center, Guangdong Provincial People's Hospital, Guangdong Academy of Medical Sciences, Guangdong Provincial Geriatrics Institute, Guangzhou, China; ^3^Clinical Laboratory, Bejing Hepingli Hospital, Beijing, China; ^4^Department of Otolaryngology-Head and Neck Surgery, Peking Union Medical College Hospital, Chinese Academy of Medical Sciences, Beijing, China; ^5^Department of Family Medicine and Division of General Internal Medicine, Department of Medicine, Peking Union Medical College Hospital, Chinese Academy of Medical Sciences, Beijing, China

**Keywords:** prevalence, multimorbidity pattern, different kinds of culture, aging, Guangdong province

## Abstract

**Background:**

Variations in the prevalence and pattern of multimorbidity might be attributable to lifestyle and environmental factors. This study was performed to determine the prevalence of common chronic diseases and to reveal multimorbidity patterns among adults in Guangdong province with Chaoshan, Hakka, and island cultures.

**Methods:**

We used data collected at the baseline survey (April–May 2021) of the Diverse Life-Course Cohort study and included 5,655 participants aged ≥20 years. Multimorbidity was defined as the presence of two or more of the 14 chronic diseases collected by self-reports, physical examinations, and blood tests. Multimorbidity patterns were explored by association rule mining (ARM).

**Results:**

Overall, 40.69% of participants had multimorbidity, and the prevalence among coastland (42.37%) and mountain residents (40.36%) was higher than that among island residents (37.97%). The prevalence of multimorbidity increased rapidly with higher age groups and showed an inflection point at 50 years, beyond which >50% of the middle-aged and older adults had multimorbidity. The proportion of people with two chronic diseases accounted for most cases of multimorbidity, and the strongest association was found between hyperuricemia and gout (lift of 3.26). The most prevalent multimorbidity pattern was dyslipidemia and hyperuricemia in the coastland areas and dyslipidemia combined with hypertension in the mountain and island areas. Furthermore, the most common triad combination consisted of cardiovascular diseases, gout, and hyperuricemia, which was verified in the mountain and coastal areas.

**Conclusion:**

These observations of multimorbidity patterns, including the most frequent multimorbidity and associations, will help healthcare providers develop healthcare plans that improve the effectiveness of multimorbidity management.

## 1. Introduction

The number of people with multiple chronic health conditions has been rising with the global population aging and the growing prevalence of non-communicable diseases (NCDs) (1–3). An estimated one-third of adults worldwide have two or more coexisting chronic diseases (4). Multimorbidity, an emerging and prominent public health issue globally, has been associated with poorer patient outcomes and poses a challenge to the increased difficulty for health systems to optimize personalized care, furthermore translating into a substantial economic burden for health systems (1, 5, 6).

A large body of literature has revealed the prevalence and patterns of multimorbidity among adults in China. The prevalence of multimorbidity ranges widely from 4.8 to 90.5% (7–10) because of variance in aspects such as geographical environments and the definitions and measurements of disease (11, 12). In general, people who are older, female, or living in northern areas have a higher prevalence of multimorbidity (12–14). As the number of older adults has grown, there has been a dramatic increase in the prevalence of multimorbidity during the past decade. According to the China Health and Retirement Longitudinal Study (CHARLS), the prevalence of multimorbidity increased from 38.6 in 2011 to 53.9% in 2018 among middle-aged and older adults, respectively (15, 16). Additionally, multimorbidity is not exclusive to older people, and it appears to affect a much broader cross-section of the population (3). A systematic review of 39 observational studies across 12 high-income countries illustrated a strong positive association between increasing age and the prevalence of multimorbidity [odds ratio (OR), 1.26–227.46] (17). Studies also have shown different multimorbidity patterns in people of different ages: multimorbidity is likely to involve mixed digestive system diseases (such as hepatobiliary disease) and other physical health conditions (such as hypertension and diabetes) in younger age groups, whereas older adults are more prone to multiple physical health conditions (18, 19). Currently, evidence on multimorbidity in younger populations is limited in China. Moreover, some studies have revealed that variations in the prevalence and pattern of multimorbidity might be attributable to lifestyle and environmental factors. However, most studies on lifestyle factors and multimorbidity have been conducted in Western countries, where the multimorbidity pattern may differ from that in the Chinese population (7, 20).

Guangdong province, a typical representative coastal area with many platforms, is located in the south of China, and its inhabitants exhibit many kinds of dietary behaviors. Chaoshan, Hakka, and Island, as typical intriguing cultures in Guangdong province, have great differences in inhabitants and lifestyle, which may lead to differences in health status (21, 22). Chaoshanese mainly reside in the Chaoshan area along the southeast coast of Guangdong province and commonly consume meats from seafood and drink hot red tea. Hakka mainly inhabit the mountains of Eastern Guangdong province, commonly consuming meats from pork and chicken and drinking green tea. Nan-Ao Island in Shantou city, which is selected as the representation of island culture, is a relatively isolated place with native residents living with unchanged customs and having porridge for three meals and fish as seasoning. Therefore, the present study was performed to determine the prevalence of common chronic diseases and reveal multimorbidity patterns among adults in Guangdong province, as well as to examine the diversity of common chronic diseases and multimorbidity patterns by different age groups and across different cultures.

## 2. Materials and methods

### 2.1. Data source

**Figure 1:**
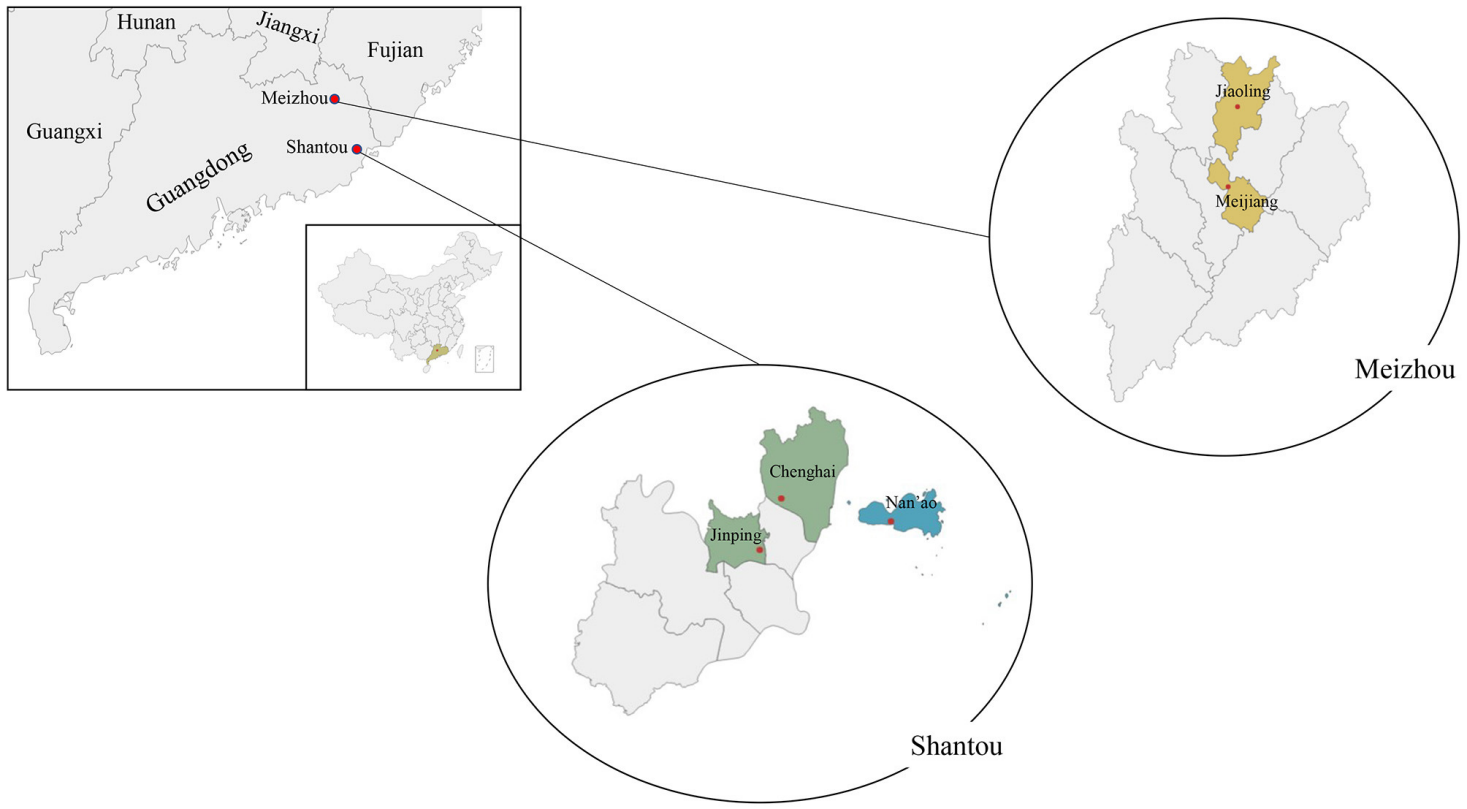
Locations of study sites in Guangdong province in the DLCC.

This study utilized the baseline data from the Diverse Life-Course Cohort (DLCC) study (23), which is a population-based prospective cohort study. The survey was conducted in Shantou and Meizhou cities in Guangdong province from April 2021 to May 2021. The locations of the study sites are shown in Figure 1. Considering the three cultures “Chaoshan”, island, and “Hakka”, we divided the study sites into coastland (including the Chenghai and Jinping districts in Shantou city), island (including Nan-Ao county in Shantou city), and mountain areas (including Meijiang district and Jiaoling county in Meizhou city).

Using a cluster random sampling method, participants aged ≥20 years who had lived in their current residence for at least 1 year were recruited in this study using the sampled units mentioned above. Individuals with severe mental or physical illness, pregnant females, and military personnel on active service were excluded. In total, 5,655 participants completed the survey; they comprised 2,419 (42.8%) coastland residents, 1,259 (22.2%) island residents, and 1,977 (35.0%) mountain area residents. All participants gave written informed consent to participate in this study. The study was approved by the Ethics Committee of the Institute of Basic Medical Sciences, Chinese Academy of Medical Sciences.

### 2.2. Data collection

Participants were invited to take a face-to-face questionnaire survey that included demographic and socioeconomic information, personal medical history, and health-related lifestyle factors such as smoking status, alcohol consumption, physical activity, and dietary patterns. The completeness and correctness of each questionnaire were examined by an epidemiologist through face-to-face re-check with the participant. This study was implemented in accordance with a previously published study (24). The participants underwent physical examinations including measurement of weight, height, blood pressure, electrocardiography, and bone mineral density. Systolic blood pressure (SBP) and diastolic blood pressure (DBP) were measured three times after at least 5 min of rest in a seated position, using a digital sphygmomanometer (Omron HEM-907, Japan). In addition, a 10-ml fasting blood sample (≥8 h) was drawn from each participant. Fasting plasma glucose (FPG, mmol/L), serum uric acid concentration (SUA, μmol/L), and serum-lipid including triglyceride (TG, mmol/L), total cholesterol (TC, mmol/L), high-density lipoprotein cholesterol (HDL-C, mmol/L), and low-density lipoprotein cholesterol (LDL-C, mmol/L) were tested in Bejing Hepingli Hospital.

### 2.3. Definition and measurement of multimorbidity

In this study, we identified 14 common chronic diseases based on the most frequently mentioned diseases for multimorbidity measures in previous studies (7, 9–11). Hypertension was defined as an average SBP of ≥140 mmHg and/or an average DBP of ≥ 90 mmHg, or self-reported hypertension history. Diabetes was defined as FPG ≥7.0 mmol/L or self-reported diabetes history. Dyslipidemia was defined as total cholesterol > 6.2 mmol/L, triglycerides ≥2.3 mmol/L, LDL-C ≥4.1 mmol/L, or HDL-C < 1.0 mmol/L or self-reported dyslipidemia history (25). Hyperuricemia was defined as SUA >420 μmol/L or self-reported hyperuricemia history (26). Other chronic diseases, such as gout, gastroenteritis/ulcer diseases (gastroenteritis, reflux esophagitis, gastric perforation, peptic ulcer disease, colitis, and rectitis), urinary system diseases (kidney stones, nephritis, renal cysts, renal insufficiency, diabetic nephropathy, and urinary tract calculi), gallbladder diseases (gallstones, cholecystitis, and gallbladder polyps), coronary heart disease, respiratory diseases (chronic obstructive pulmonary disease, asthma, bronchitis, emphysema, and pulmonary nodules), cancer, stroke, arthritis or rheumatism, and liver diseases (hepatitis, cirrhosis, and hepatic cysts) were identified according to self-reported physician professional diagnosis. All diseases were defined as a binary variable (yes or no). Multimorbidity was defined as two or more coexisting chronic conditions within one person.

### 2.4. Statistical analysis

Age as a continuous variable is presented as mean ± standard deviation and median (P_25_, P_75_). Overall prevalence was calculated as the number of cases divided by all included participants in the sample and age-specific prevalence; the Wilson method was used to estimate the prevalence of multimorbidity and its 95% confidence interval (CI). Other categorical variables are summarized as count (percentage), and the chi-square test was performed to compare these characteristics. Direct standardization was performed using China's population age structure from the China Statistical Yearbook 2021.

Association rule mining (ARM) was used to determine common multimorbidity patterns. Association rules were relationships between sets of diseases from itemset1 (called “Conditions”) to itemset2 (called “Outcomes”). Support, confidence, and lift in particular were used as measurement indicators in ARM, in which the support is equivalent to the prevalence of disease combinations; the confidence is the conditional probability of occurrence of itemset2, given itemset1; a higher value of lift (>1) indicates that the relationship between itemset1 and itemset2 is more significant than expected if the two were independent. This further suggests that itemset1 is positively associated with itemset2 (27). For this study, we first used an *a priori* algorithm with a threshold of support of 0.001 (confidence was not limited) to identify all critical association rules, in which the percentage in the whole dataset that contains both conditions and outcomes is no < 0.1%. Then, to identify more frequent and potentially important patterns, the minimum thresholds of the parameters in ARM were defined as follows: support >0.5%, confidence >30%, and lift >1. The function “apriori” in the R package “arules” was used in the clustering analysis. All the analyses were performed using the SAS 9.4 software (SAS Institute Inc., Cary, NC, USA) and R4.1.2 (R Development Core Team).

## 3. Results

### 3.1. Sample description

The general characteristics of the 5,655 study participants are shown in Table 1. The mean age was 52.9 ± 12.91 years, the majority of participants were in the 40–69 age group (74.45%), and 1,660 (29.35%) were men. Significant differences among the three groups were seen in all characteristics except alcohol drinking. Coastland residents showed higher education and personal annual income, island residents were older and comprised more women, and mountain residents had a higher body mass index and physical activity and a lower incidence of smoking behavior.

**Table 1:** Characteristics of participants recruited from different regions.

	**Total**	**Island**	**Mountain**	**Coastland**	***P*-value for three different regions**
*N*	5,655	1,259	1,977	2,419	
**Age, yr**					
Mean (sd)	52.9 (12.91)	54.0 (11.92)	53.7 (12.19)	51.7 (13.83)	< 0.0001
Medium (P_25_, P_75_)	54 (45, 62)	55 (48, 63)	54 (46, 62)	54 (41, 62)	< 0.0001
**Age group, yr**, ***n*** **(%)**					< 0.0001
20–29	306 (5.41)	59 (4.69)	58 (2.93)	189 (7.81)	
30–39	636 (11.25)	87 (6.91)	212 (10.72)	337 (13.93)	
40–49	1,128 (19.95)	247 (19.62)	415 (20.99)	466 (19.26)	
50–59	1,799 (31.81)	476 (37.81)	665 (33.64)	658 (27.20)	
60–69	1,283 (22.69)	281 (22.32)	438 (22.15)	564 (23.32)	
≥70	503 (8.89)	109 (8.66)	189 (9.56)	205 (8.47)	
**Sex**, ***n*** **(%)**					< 0.0001
Male	1,660 (29.35)	297 (23.59)	577 (29.19)	786 (32.49)	
Female	3,995 (70.65)	962 (76.41)	1,400 (70.81)	1,633 (67.51)	
**BMI group**^a^, ***n*** **(%)**					0.0002
Underweight	291 (5.16)	64 (5.09)	77 (3.90)	150 (6.24)	
Normal	2,964 (52.59)	674 (53.62)	990 (50.15)	1,300 (54.05)	
Overweight	1,861 (33.02)	415 (33.02)	704 (35.66)	742 (30.85)	
Obesity	520 (9.23)	104 (8.27)	203 (10.28)	213 (8.86)	
**Education attainment**, ***n*** **(%)**					< 0.0001
Primary school or below	1,370 (24.26)	588 (46.85)	265 (13.42)	517 (21.39)	
Middle school	2,966 (52.53)	457 (36.41)	1,268 (64.24)	1,241 (51.34)	
High school and above	1,310 (23.20)	210 (16.73)	441 (22.34)	659 (27.27)	
**Personal annual income (RMB)**, ***n*** **(%)**					< 0.0001
< 10,000	434 (7.78)	124 (9.86)	165 (8.38)	145 (6.16)	
10,000–29,999	2,122 (38.04)	584 (46.46)	847 (43.02)	691 (29.38)	
30,000–49,999	1,555 (27.88)	286 (22.75)	426 (21.64)	843 (35.84)	
≥50,000	1,467 (26.30)	263 (20.92)	531 (26.97)	673 (28.61)	
**Smoking**, ***n*** **(%)**					< 0.0001
Never	4,624 (81.78)	1,044 (82.92)	1,733 (87.66)	1,847 (76.39)	
Ever	211 (3.73)	25 (1.99)	84 (4.25)	102 (4.22)	
Current	819 (14.49)	190 (15.09)	160 (8.09)	469 (19.40)	
**Drinking**, ***n*** **(%)**					0.1363
Never	4,717 (83.44)	1,073 (85.23)	1,620 (81.94)	2,024 (83.74)	
Ever	98 (1.73)	22 (1.75)	34 (1.72)	42 (1.74)	
Current	838 (14.82)	164 (13.03)	323 (16.34)	351 (14.52)	
**Physical activity**^b^, ***n*** **(%)**					< 0.0001
Low	1,517 (26.84)	509 (40.43)	303 (15.33)	705 (29.17)	
Moderate	681 (12.05)	160 (12.71)	210 (10.62)	311 (12.87)	
High	3,455 (61.12)	590 (46.86)	1,464 (74.05)	1,401 (57.96)	

BMI, body mass index.

^a^Underweight was defined as a BMI of < 18.5 kg/m^2^, normal weight as a BMI of 18.5 to < 24.0 kg/m^2^, overweight as a BMI of 24.0 to < 28.0 kg/m^2^, and obesity as a BMI of ≥28.0 kg/m^2^.

^b^The level of physical activity, considered both occupational and leisure-time physical activity, was classified as low (light level of both occupational and leisure-time physical activity), moderate (moderate or high level of either occupational or leisure-time physical activity), or high (moderate or high level of both occupational and leisure-time physical activity).

### 3.2. Prevalence of chronic diseases

**Figure 2:**
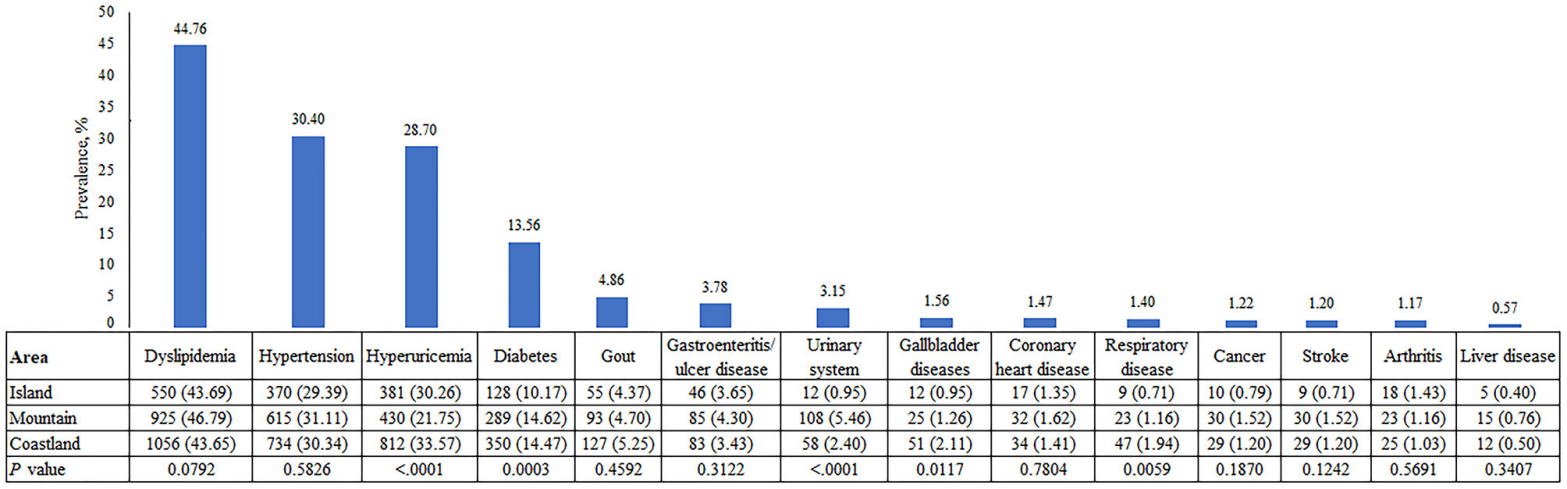
The observed prevalence of chronic diseases by region.

Figure 2 shows the prevalence of chronic diseases among the survey participants. Dyslipidemia was the most frequent disease, with a prevalence as high as 44.76%, far higher than the other chronic diseases. The prevalence of other chronic diseases, in descending order, was 30.40% for hypertension, 28.70% for hyperuricemia, 13.56% for diabetes, and 4.86% for gout. The pattern was similar between coastland and island residents, except that the fifth disease was urinary system diseases in the mountain area residents. Furthermore, significant differences among the three areas were seen in hyperuricemia, diabetes, urinary system diseases, gallbladder diseases, and respiratory diseases. Hyperuricemia was significantly higher among coastland and island residents (>30.0%) than among mountain residents (21.75%), island residents had the lowest prevalence rate of diabetes (10.17%), the rate of urinary system diseases in mountain area residents was higher than that in coastland and island residents (5.46 vs. 2.40 and 0.95%, respectively), and coastland residents had a higher prevalence of gallbladder diseases and respiratory diseases.

### 3.3. Prevalence and pattern of chronic multimorbidity

**Figure 3:**
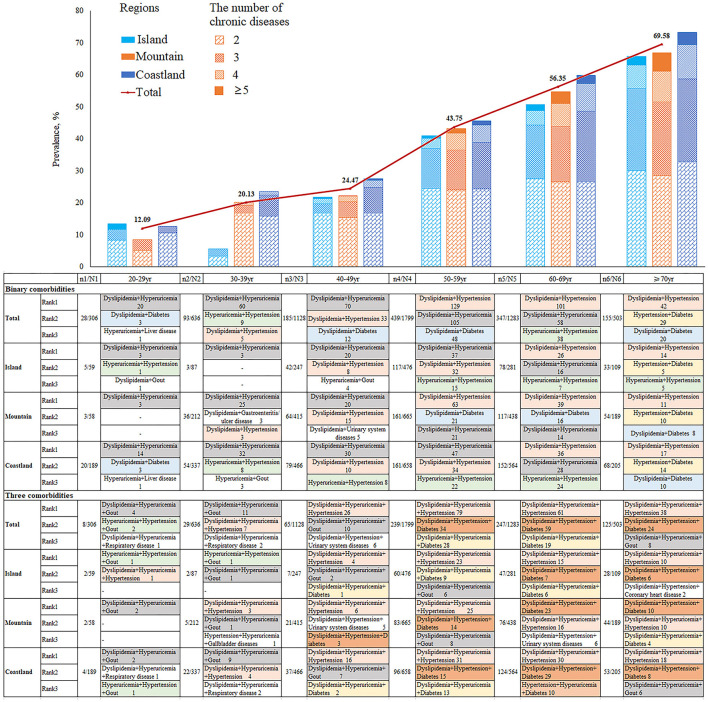
The top binary and ternary multimorbidity combinations by age and region.

The burden of multimorbidity is presented in Figure 3. The prevalence of multimorbidity among the population was 40.69%, which significantly increased with age from 12.09% in the 20–29 years age group to 69.58% in the ≥70-year age group. Of participants aged >60 years, 60% had multimorbidity. Similar trends were found in different areas; there was a significant difference in the distribution of multimorbidity among the three regions (37.97, 40.36, and 42.37% in island, mountain, and coastland areas, respectively). Moreover, the age-standardized rate was 41.90% (95% confidence interval: 39.66–44.13%) using the population census data of China in 2020 as the reference population. The standardized rates were different in the coastland [43.40% (40.23–46.57%)], mountain [41.56% (37.31–45.81%)], and island areas [36.47% (31.41–41.53%)].

Among the 2,301 participants with multimorbidity, 1,247 (54.2%) had two diseases. The top three binary multimorbidity combinations were dyslipidemia and hyperuricemia (26.22%, 327/1247), dyslipidemia and hypertension (24.86%, 310/1247), and hyperuricemia and hypertension (9.46%, 118/1247). A total of 713 (31.0%) participants had three diseases. The top ternary multimorbidity combinations were dyslipidemia, hyperuricemia, and hypertension; dyslipidemia, hypertension, and diabetes; and dyslipidemia, hyperuricemia, and diabetes. In addition, 256 (11.1%) and 85 (3.7%) participants had four diseases and five or more diseases, respectively. Figure 3 shows the top binary and ternary multimorbidity combinations by age and region. For the binary multimorbidity groups, dyslipidemia and hyperuricemia were the most common combination in young and middle-aged participants (< 50 years of age), and dyslipidemia and hypertension were the most common combination in the older groups. Unlike in the mountain area (20.7%, 90/435), dyslipidemia combined with hyperuricemia was the most common binary combination in the coastland (29.4%, 157/534) and island regions (28.8%, 80/278). For the ternary multimorbidity combination groups, dyslipidemia, hyperuricemia, and hypertension were the most common disease combination, especially in middle-aged and older participants. Furthermore, the combination of dyslipidemia, hypertension, and diabetes was more common in older groups.

### 3.4. ARM results

The associations among the included diseases were illustrated by ARM. To avoid missing any critical association rules, 434 rules were generated after running the *a priori* algorithm based on the threshold of support of 0.001 at the beginning (confidence was not limited). A total of 72 patterns were finally kept through threshold filtering. The top 10 association rules among these diseases according to lift are presented in Tables 2A, B. For the binary multimorbidity groups, the strongest association was found between the combination of hyperuricemia and gout, for which the lift was 3.26. That means gout is positively associated with hyperuricemia. Meanwhile, the rule “gout”-> “hyperuricemia” showed that individuals who have gout are most likely to experience hyperuricemia (93.45% probability). The support indicated that these two diseases co-exist in 4.54% of the participants within a certain period. Even if the lift values were different, a similar pattern of hyperuricemia and gout was shown in three areas. Among the coastland area participants, the second strongest association was observed between diabetes and coronary heart disease, for which the lift was 2.64, whereas the second lift for the island and mountain areas was 2.40 and 2.43 for the combination of hypertension and coronary heart disease and the combination of diabetes and gout, respectively. Moreover, hypertension tended to be comorbid with other diseases that occurred in coastland participants; dyslipidemia tended to be comorbid with other diseases that occurred in island participants; and patients with stroke or coronary heart disease tended to have a higher risk of hypertension, hyperuricemia, and diabetes among mountain areas.

**Table 2A:** Top 10 association rules in order of lift with dyads of morbidities.

**Total**					**Island**				
**Conditions**	**Outcomes**	**Support, %**	**Confidence, %**	**Lift**	**Conditions**	**Outcomes**	**Support, %**	**Confidence, %**	**Lift**
Gout	Hyperuricemia	4.54	93.45	3.26	Gout	Hyperuricemia	3.89	89.09	2.94
Coronary heart disease	Hypertension	1.01	68.67	2.26	Coronary heart disease	Hypertension	0.95	70.59	2.40
Stroke	Hypertension	0.78	64.71	2.13	Urinary system diseases	Hyperuricemia	0.56	58.33	1.93
Diabetes	Hypertension	7.64	56.32	1.85	Diabetes	Hypertension	5.00	49.22	1.67
Stroke	Hyperuricemia	0.57	47.06	1.64	Coronary heart disease	Dyslipidemia	0.95	70.59	1.62
Gout	Dyslipidemia	3.34	68.73	1.54	Gout	Hypertension	1.99	45.45	1.55
Gout	Hypertension	2.23	45.82	1.51	Urinary system diseases	Dyslipidemia	0.64	66.67	1.53
Coronary heart disease	Hyperuricemia	0.62	42.17	1.47	Gout	Dyslipidemia	2.86	65.45	1.50
Diabetes	Dyslipidemia	8.79	64.8	1.45	Arthritis	Hyperuricemia	0.64	44.44	1.47
Coronary heart disease	Dyslipidemia	0.94	63.86	1.43	Diabetes	Dyslipidemia	6.43	63.28	1.45
**Mountain**					**Coastland**				
**Conditions**	**Outcomes**	**Support, %**	**Confidence, %**	**Lift**	**Conditions**	**Outcomes**	**Support, %**	**Confidence, %**	**Lift**
Gout	Hyperuricemia	4.35	92.47	4.25	Gout	Hyperuricemia	5.04	96.06	2.86
Gout	Diabetes	1.67	35.48	2.43	Coronary heart disease	Diabetes	0.54	38.23	2.64
Stroke	Diabetes	0.51	33.33	2.28	Stroke	Hypertension	0.83	68.97	2.27
Coronary heart disease	Hypertension	1.11	68.75	2.21	Coronary heart disease	Hypertension	0.95	67.65	2.23
Coronary heart disease	Diabetes	0.51	31.25	2.14	Diabetes	Hypertension	8.52	58.86	1.94
Stroke	Hypertension	0.96	63.33	2.04	Stroke	Hyperuricemia	0.66	55.17	1.64
Stroke	Hyperuricemia	0.66	43.33	1.99	Gout	Dyslipidemia	3.72	70.87	1.62
Diabetes	Hypertension	8.24	56.4	1.81	Gout	Hypertension	2.32	44.09	1.45
Coronary heart disease	Hyperuricemia	0.61	37.5	1.72	Diabetes	Dyslipidemia	9.01	62.29	1.43
Respiratory disease	Hypertension	0.61	52.17	1.68	Urinary system diseases	Hypertension	1.03	43.1	1.42

**Table 2B:** Top 10 association rules in order of lift with triads of morbidities.

**Total**					**Island**				
**Conditions**	**Outcomes**	**Support, %**	**Confidence, %**	**Lift**	**Conditions**	**Outcomes**	**Support, %**	**Confidence, %**	**Lift**
Hypertension + Gout	Hyperuricemia	2.16	96.83	3.37	Hyperuricemia + Coronary heart disease	Hypertension	0.56	100.00	3.40
Dyslipidemia + Gout	Hyperuricemia	3.22	96.3	3.36	Hypertension + Gout	Hyperuricemia	1.91	96.00	3.17
Diabetes + Gout	Hyperuricemia	1.06	95.24	3.32	Dyslipidemia + Gout	Hyperuricemia	2.70	94.44	3.12
Coronary heart disease + Dyslipidemia	Hypertension	0.64	67.92	2.23	Dyslipidemia + Coronary heart disease	Hypertension	0.64	66.67	2.26
Hypertension + Gout	Diabetes	0.67	30.16	2.22	Hypertension + Gastroenteritis/ulcer disease	Hyperuricemia	0.56	63.64	2.10
Diabetes + Gout	Hypertension	0.67	60.32	1.98	Hypertension + Coronary heart disease	Hyperuricemia	0.56	58.33	1.93
Diabetes + Hyperuricemia	Hypertension	3.06	59.66	1.96	Hypertension + Gastroenteritis/ulcer disease	Dyslipidemia	0.71	81.82	1.87
Diabetes + Dyslipidemia	Hypertension	4.97	56.54	1.86	Diabetes + Hyperuricemia	Dyslipidemia	3.10	79.59	1.82
Diabetes + Gout	Dyslipidemia	0.87	77.78	1.74	Diabetes + Hyperuricemia	Hypertension	2.07	53.06	1.81
Diabetes + Hyperuricemia	Dyslipidemia	3.93	76.55	1.71	Diabetes + Dyslipidemia	Hypertension	3.18	49.38	1.68
**Mountain**					**Coastland**				
**Conditions**	**Outcomes**	**Support,%**	**Confidence, %**	**Lift**	**Conditions**	**Outcomes**	**Support,%**	**Confidence, %**	**Lift**
Diabetes + Hyperuricemia	Gout	1.62	31.07	6.60	Dyslipidemia + Gout	Hyperuricemia	3.68	98.89	2.95
Diabetes + Gout	Hyperuricemia	1.62	96.97	4.46	Hypertension + Gout	Hyperuricemia	2.27	98.21	2.93
Hypertension + Gout	Hyperuricemia	2.18	95.56	4.39	Diabetes + Gout	Hyperuricemia	0.95	95.83	2.85
Dyslipidemia + Gout	Hyperuricemia	2.98	93.65	4.31	Hypertension + Dyslipidemia	Diabetes	5.17	32.05	2.22
Dyslipidemia + Stroke	Hyperuricemia	0.51	66.67	3.07	Diabetes + Hyperuricemia	Hypertension	3.35	58.70	1.93
Hypertension + Gout	Diabetes	1.01	44.44	3.04	Diabetes + Dyslipidemia	Hypertension	5.17	57.34	1.89
Dyslipidemia + Gout	Diabetes	1.32	41.27	2.82	Diabetes + Gout	Hypertension	0.54	54.17	1.79
Hyperuricemia + Coronary heart disease	Hypertension	0.51	83.33	2.68	Diabetes + Hyperuricemia	Dyslipidemia	4.34	76.09	1.74
Hyperuricemia + Urinary system diseases	Diabetes	0.61	37.5	2.57	Diabetes + Gout	Dyslipidemia	0.74	75.00	1.72
Hyperuricemia + Gout	Diabetes	1.62	37.21	2.55	Hyperuricemia + Gout	Dyslipidemia	3.68	72.95	1.67

Among ternary multimorbidity (shown in Table 2B), the strongest association was found for the combination of diabetes, hyperuricemia, and gout, for which the lift was 3.37. This means that the chance of these diseases occurring together was 3.37 times higher than would be expected if they were independent. Furthermore, diabetes and gout appeared most in the six rules as conditions and seemed to have a strong relationship with hyperuricemia, hypertension, and dyslipidemia. Among different areas, the highest lift of 6.60 was observed for the combination of diabetes, hyperuricemia, and gout in the mountain area; the combination of dyslipidemia, hyperuricemia, and gout in the coastland area (lift = 2.95); and the combination of hypertension, hyperuricemia, and coronary heart disease in the island area (lift = 3.40). Dyslipidemia, hyperuricemia, gout, diabetes, and hypertension were the most common multimorbidity.

## 4. Discussion

In this study, we showed that multimorbidity was prevalent in Guangdong province. There was a significant difference in the distribution of multimorbidity among three different regions: coastland residents (42.37%) had a higher prevalence than mountain residents (40.36%) and island residents (37.97%). Hyperuricemia and gout, in addition to dyslipidemia, hypertension, and diabetes, were identified as the most prevalent diseases. We also showed that the prevalence of multimorbidity increased in populations of higher age groups, showing an inflection point at 50 years, beyond which >50% of the middle-aged and older adults had multimorbidity. The multimorbidity pattern involving two chronic diseases changed from dyslipidemia and hyperuricemia to dyslipidemia combined with hypertension with increasing age. Furthermore, the most common triad combination consisted of cardiovascular diseases, gout, and hyperuricemia, which was verified in the mountain and coastal areas.

We found that the prevalence of multimorbidity was 40.69% in our sample from three survey areas in Guangdong province, which is consistent with the range of the overall prevalence in Guangdong province (9, 28, 29). One finding in this study that is worthy of particular attention is the regional difference: the prevalences among coastland and mountain residents were higher than those among island residents. An explanation is that information regarding the participants' medical history was obtained through self-reports and supplemented by physical examinations or blood tests. Considering the long-term traffic blockages and insufficient medical resources, health literacy is inadequate in the island population. For example, the awareness of hypertension was only 50% in the island region, < 58% in the coastland area, and 60% in the mountain area. The current study findings may have important implications that government should strengthen medical resource allocation for island areas, which may be closely related to multimorbidity prevention and control. With respect to the number of morbidities, the proportion of people with two chronic diseases accounted for most participants with multimorbidity, and the proportion was highest in the island area (58%). Regarding specific compositions of multimorbidity, the most prevalent multimorbidity pattern was dyslipidemia and hyperuricemia in the coastland area and dyslipidemia and hypertension in the mountain and island areas. One alternative explanation might be that hypertension was more common than hyperuricemia in the mountain areas (31.11 vs. 21.75%); the population surveyed in isolated island areas was older, and age is reportedly the major risk factor for cardiovascular diseases (30). Additionally, differences in population and socioeconomic status may have interfered with the comparison of regional effects among different studies. Thus, the strategies for the prevention and control of chronic diseases should be tailored to different regions. Previous studies have shown that residents of the coastal area have poor dietary habits (such as excessive alcohol drinking, seafood intake, and midnight snack), which can lead to metabolic disorders (31). The government should increase the promotion of health education. For island areas with a large proportion of the older adults population, the control level of chronic diseases should be actively monitored to improve residents' compliance with follow-up visits.

We found that the prevalence of multimorbidity was as low as 12.09% for young people aged 20–29 years and as high as 69.58% for older adults aged ≥70 years. Although older adults had a higher prevalence of multimorbidity, which is consistent with previous studies, multimorbidity was not unique to older adults (6, 18). Our results suggest an inflection point in the prevalence of multimorbidity at 50 years, beyond which more than 50% of the middle-aged and older adults had multimorbidity. This is consistent with the result from the CHARLS in 2018 (16). Furthermore, the prevalence found in our study is commensurate with the ranges found in other countries: the index among adults aged ≥65 years was 63.7% in the United States (32) and 62.8% in Japan (33). For the multimorbidity patterns, dyslipidemia combined with hyperuricemia was the most common pattern among young people, while dyslipidemia combined with hypertension or diabetes was identified among the advanced-age population; this is consistent with natural aging-related changes (30). This phenomenon indicates that the management of multimorbidity should be age-specific. In particular, young people are more likely to have modifiable lifestyle factors preventing the incidence of metabolic disorders, (20, 34) whereas older persons with long-existing multimorbidity should receive increased resources and policy input, and the focus of health system services should be adjusted to improve the effectiveness of multimorbidity management (5, 35, 36).

At present, there is no international consensus regarding the best way to define and measure multimorbidity (37). Hyperuricemia and gout, the most prevalent diseases in Guangdong province, were included. Our study showed that the prevalences of hyperuricemia and gout were 28.70 and 4.86%, respectively, which were higher than the pooled prevalences of gout in the adult population in China (13.3 and 1.1%, respectively) (38) and consistent with previous studies in Guangdong province (39). The main reasons for this phenomenon are closely related to the local characteristics of residents' eating habits. Residents prefer broth in hot and humid weather, which would contain too many purines. For example, nearly 25% of people eat the local special food “Saam Kap Dai” every week, which consists of pork, chitterlings, and belly. In addition, the prevalence of hyperuricemia was higher in the island (30.26%) and coastal regions (33.57%) with *p* < 0.0001, which may be due to the consumption of seafood, and the frequency of weekly seafood consumption was much higher in the coastland (95%) and island (97%) areas than in the mountain areas (47%). Furthermore, a strong association between hyperuricemia and gout was detected by ARM in all regions, which is in accordance with the fact that hyperuricemia is the most dominant factor in gout development (26, 40). Moreover, our study showed that patients with coronary heart disease and/or stroke were prone to occur hyperuricemia, and the connections between diseases have been recognized by mass epidemiological data (41–43).

We found that the most prevalent disease pair was dyslipidemia combined with hyperuricemia and that the most common triad combination was dyslipidemia, hyperuricemia, and hypertension. Considering the differences in the definitions and measurements of multimorbidity, this multimorbidity pattern was initially revealed based on the specificity of the study population. Moreover, the lift in ARM revealed interesting combinations of multimorbidity that occurred more frequently than expected (27, 44). In our study, the ranked lift indicated strong associations between cardiovascular disease and metabolic diseases, including coronary heart disease, stroke, and hypertension with hyperuricemia, diabetes, and dyslipidemia. In agreement with our findings, the pathophysiological connections between diseases in these chronic disease pairs are well-recognized (45–47). Through the above analysis, the frequency of different diseases in different multimorbidities can be identified, and the focus of community multimorbidity prevention and control can be clarified. The level of chronic disease multimorbidity in the community can be significantly reduced through the prevention and control of such key chronic diseases, which will greatly improve the efficiency of community-level chronic disease prevention.

Multimorbidity is common in the surveyed areas, thus clinicians, especially family doctors, need to take into consideration the overall health conditions of patients with multiple chronic diseases to satisfy patient-specific needs regarding disease diagnosis and treatment. They should actively explore multiple approaches to implement polypharmacy management for adults with multimorbidity, particularly problems of polypharmacy in older adults. Considering the importance of primary care in addressing multimorbidity, strengthened training of primary-level medical workers is required to better prevent and control NCDs and to deliver multimorbidity care in a coordinated and continuous manner. Our findings on the multimorbidity patterns in different areas could help formulate more reasonable public health policies to maximize the benefits of medical services.

The main strength of this study was the large sample size of the population-based data used to illustrate the multimorbidity prevalence and patterns among three areas in Guangdong province. However, the study also had some limitations. First, self-report bias might exist because we included self-reported information. Therefore, the prevalence of multimorbidity might be underestimated, and factors associated with multimorbidity patterns should be interpreted with caution because of the problem of underdiagnosis. Second, selection bias might exist because we did not include individuals who were unable or unwilling to participate in this survey. Third, although the survey was based on the DCLL study, the cross-sectional data of the current study made it impossible to confidently draw causal conclusions. Fourth, the definition of multimorbidity should be further improved considering the limited number and types of diseases included. Our list was not exhaustive and included 14 common NCDs, and we may have overlooked some other higher-burden conditions. This may have led to an underestimation of the prevalence and impact of multiple diseases. In addition, the areas included in our study have unique cultures, dietary habits, and geography, thus the results may not be extrapolated to other areas in China.

In conclusion, with a prevalence approaching 40%, multimorbidities were common among adults aged ≥20 years in Guangdong province, and the prevalence increased with age. Coastland residents with “Chaoshan” culture had a higher prevalence than other areas. The most prevalent disease pair was dyslipidemia combined with hyperuricemia. The most common triad combination was dyslipidemia, hyperuricemia, and hypertension. Cardiovascular diseases and metabolic diseases were more likely to co-occur. These findings in our study, including the most frequent multimorbidity, associations, and clusters, will help healthcare providers to develop healthcare plans that will improve the effectiveness of multimorbidity management.

## Data availability statement

The raw data supporting the conclusions of this article will be made available by reasonable request by sending email to the corresponding author. Requests to access the datasets should be directed to GS, guangliang_shan@163.com.

## Ethics statement

The study was approved by the Ethics Committee of Institute of Basic Medical Sciences, Chinese Academy of Medical Sciences. The patients/participants provided their written informed consent to participate in this study.

## Author contributions

GS, QO, LP, XZ, and XC contributed to the study conception and design. YH and HH performed the data analysis. The first draft of the manuscript was written by YH. All authors contributed to the material preparation, data collection, interpreted the results, revised, and approved the final manuscript.
